# Cross-cultural adaptation and psychometric properties of the Indonesian version for quality of life among breast cancer patients

**DOI:** 10.3389/fpubh.2023.1069422

**Published:** 2023-02-28

**Authors:** Solikhah Solikhah, Dyah Aryani Perwitasari, Dwi Sarwani Sri Rejeki

**Affiliations:** ^1^Faculty of Public Health, Universitas Ahmad Dahlan, Yogyakarta, Indonesia; ^2^Faculty of Pharmacy, Universitas Ahmad Dahlan, Yogyakarta, Indonesia; ^3^Department of Public Health, Faculty of Health Sciences, Universitas Jenderal Soedirman, Purwokerto, Indonesia

**Keywords:** breast cancer, Indonesian EORTC QLQ-BR45, quality of life, validation, psychometric properties

## Abstract

**Background:**

Breast cancer is one of the most important health problems worldwide. Quality of life (QoL) is an important indicator to evaluate symptoms in cancer patients, including those with breast cancer. Culturally suitable, valid, reliable, and appropriate instruments to measure the QoL of breast cancer patients are needed, which is still rare in Indonesia. This study aimed to translate the EORTC QLQ-BR45 instrument into Indonesian and evaluate its psychometrics.

**Methods:**

A cross-sectional study was performed on 635 patients conveniently selected from the oncology department in referral hospital. The first phase of this study involved translation of the existing EORTC QLQ-BR45 into Indonesian, and in the second phase, we evaluated its psychometric properties. Construct validity was evaluated using confirmatory factor analysis (CFA). Criterion validity was examined according to the association between disease stage and Karnofsky Performance Scale (KPS).

**Results:**

A total of 635 (99.00%) completed the EORTC QLQ-BR45 successfully. The instrument indicated good readability and high content validity. All Cronbach's alpha coefficients were satisfactory (overall value, 0.87). For construct validity, patients with KPS ≥80% did better than those with KPS ≤70% as did two multi-item scales in functional scales (body image and breast satisfaction) and five multi-item scales in symptom scales (systemic therapy side effects, endocrine therapy, and arm, breast, and endocrine sexual symptoms). Body image score of late-stage patients was significantly higher. CFA indicated that the nine-factor structure of the Indonesian EORTC QLQ-BR45 was a good fit for the data.

**Conclusion:**

The Indonesian EORTC QLQ-BR45 questionnaire is reliable and valid with good psychometric properties, thus can be used for breast cancer patients in Indonesia.

## Background

The GLOBOCAN data stated that 9.3 million people are living with cancer in 2020 (1), with an estimated increase to 28.4 million in 2040, increasing 47% from 2020. Breast cancer is the most common cancer found in women globally and is the fifth leading cause of cancer death worldwide. However, the most striking fact is that nearly 70% of the total cancer deaths occur in low and middle-income countries (LMICs) (2). In Indonesia, breast cancer (16.6%) is the most common type of cancer and ranks second as the cause of cancer deaths (9.6%) after lung cancer (13.2%) (3). More importantly, breast cancer-related deaths are often linked to the fact that patients are diagnosed when the disease already progresses into an advanced stage (4), which seriously compromises treatment options and results in a poor prognosis (5).

Advances in medical technology for cancer screening and cancer treatment (6), including palliative care, have resulted in more cancer survivors living longer, including breast cancer survivors (7–10). In general, breast cancer survivors often suffer from various symptoms related to long-term disease and treatment, with the accumulation of psychological distress, sexual problems, cognitive disruption, economic problems, and disease-related symptoms (fatigue, chronic pain, etc.) (11, 12). More specifically, the psychological distress has been linked to the suicidal idea among breast cancer survivors (13). These lead to impaired quality of life (QoL) of these survivors (14, 15) and their family members (16).

The Quality of Life (QoL) is an individual's perspective on life based on his or her current circumstances (17, 18). Aside from this subjective aspect, QoL instruments are highly valid, reliable, and responsive for measuring important clinical changes and comparing different types and levels of diseases, treatments, or interventions in different situations at a specific time (19, 20). A previous study indicated the evaluating adjuvant therapy treatments in 530 cancer patients treated with tamoxifen experience side effects in their emotional, functional, and sexual functions that are manifested in, among others, sleep disturbances, hot flushes, vaginal dryness, and depression (21). Also, based on a previously survey of 902 patients, treatment using aromatase inhibitors for breast cancer patients is better tolerated than tamoxifen. Unfortunately, the side effects of this treatment are slightly more severe than the side effects of tamoxifen and include bone loss, and insomnia (22). Women who have undergone cancer medication have physical and psychological effects that affect the their QoL. To date, researchers have also developed new procedures in breast cancer reconstruction techniques, which affect the QoL (23). In addition, the QoL in general can also measure the perceived disease burden that can inform the health care workers in selecting the approaches to be used in patient empowerment, and is useful in interpreting clinical outcomes and making decisions for treatment (24–26). Hence, the availability of standard and valid instruments to assess the quality of life in breast cancer patients during treatment is crucial to designing and implementing suitable interventions for breast cancer prevention.

Several instruments have been developed to measure the QoL in the oncological setting, which cover both general quality of life and specific QoL for cancer diseases like breast cancer (27, 28). Since 1993, the European Organization for Research and Treatment of Cancer (EORTC) has established an integrated framework for assessing the QoL among any type of cancer patients, including the instrument specific of breast cancer, called the EORTC QLQ-BR23. It is comprised of 23 items and has been rendered into more than 60 different languages. One of the first modules made to go with the core questionnaire, the EORTC QLQ-C30, was the EORTC QLQ-BR23 (29). Since 1996, breast cancer detection and therapy have improved significantly, hence the previous EORTC QLQ-BR23 was deemed inadequate to assess several essential QoL concerns, including probable side effects of newer treatments. Aromatase inhibitors, such as tamoxifen, which was once the gold standard for hormonal breast cancer therapy in postmenopausal women, can cause toxicities such as arthralgia, bone loss, and cognitive dysfunction (30–32). Then, in the last 10 years, chemotherapy treatment was expanded to include a taxane-based treatment regimen as new standard adjuvant treatment agents for early breast cancer. The quality of life of breast cancer patients is profoundly affected by the adverse effects of these chemotherapeutic drugs (33, 34). Additionally, new surgical technique was having unanticipated repercussions on patients' quality of life (23). Due to the fact that the EORTC QLQ-BR23 considerably underreports each of these side effects, the EORTC Quality of Life Group revised the questionnaire into the EORTC QLQ-BR45. It has 45 items, 23 of which are from the original QLQ-BR23, and has been translated into 19 languages (35). The English version of this questionnaire has been tested and found to be valid in Western countries (36, 37); however, no specific validation and translation for Asian countries, including Indonesia, has been completed. To be able to translate the instrument into a good instrument in the target language, strategies should be applied to increase the accuracy of semantic equivalence with the target population, as well as to increase the conceptual accuracy where the concept of the source text is conveyed accurately and by taking the norms and culture of target population into account (38, 39). Quality of life (QoL) is an important end point in medical and health research (40) which represents an individual's perception of their life in society in the context of the existing culture and norms and is related to their goals, expectations, standards, and concerns. Breast cancer patients are experiencing changes in their physical, psychological (depression and anxiety), and social aspects, as well as in their sexual function and daily activities. These changes significantly influence the quality of life (41). Hence, the availability of a specific, standardized, and valid instruments to assess the quality of life of breast cancer patients during treatment is crucial to design and implement suitable interventions for breast cancer patients (42–44). Moreover, more than 50 percent of breast cancer patients delay seeking for medical treatment and fear of mastectomy has been stated as one of their reasons for delaying treatment (45). In another study, 113 Indonesian breast cancer survivors mentioned that they experience fatigue, anxiety, and depression (46). Therefore, this study aimed to adapt and evaluate the psychometric properties of the Indonesian version of EORTC QLQ-BR45 for breast cancer patients.

## Methods

### Study design

**Figure 1:**
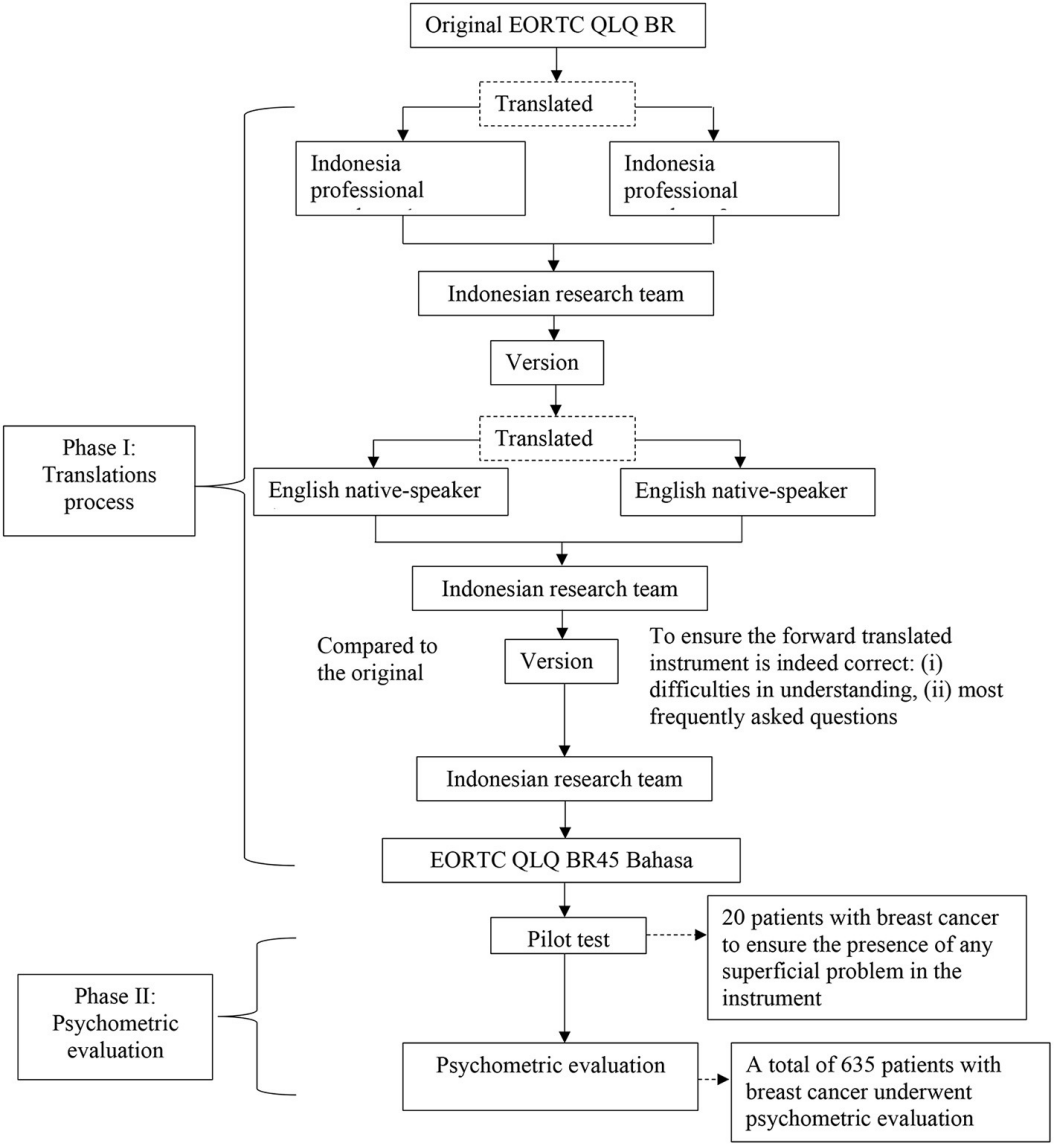
Multiple standardized process flowchart of this study.

This study used a cross-sectional design. Data collection was performed from July to September 2021 after receiving the ethical clearance from the Research Ethics Committee of Ahmad Dahlan University, Yogyakarta, Indonesia with the issuance of the ethical clearance number 012102016 on April 28, 2021. All participants in this study verbally gave their consent after receiving information on the study and were informed that they could withdraw their voluntary participation at any time. This two-phase study was designed to evaluate the psychometric properties of the Indonesian EORTC QLQ-BR45. The first stage involved translation of the original English version of the questionnaire into the Indonesian version, which was then followed by validation of the translation. The second stage involved the evaluation of the psychometric properties of the Indonesian EORTC QLQ-BR45. Figure 1 presents the flow of this study.

### Phase 1: Instrument translation and face validity

The EORTC QLQ-BR45 for breast cancer survivors was translated from the original English version into the simplified Indonesian version. The procedures for translation into the Indonesian and the forward-backward translation techniques were performed according to the recommendations in international guidelines (47, 48). First, the questions from the original version of the EORTC QLQ-BR45 were translated into Indonesian by two independent bilingual translators. Then, to get the first version in Indonesian, it was reviewed by a group of oncologists and epidemiologists on a professional committee regarding linguistic, semantic and contextual aspects (49). The final version created in this step was labeled “version 1.” In the backward translation process, the version 1 document was independently translated from Indonesian to English by two native speakers of English, who had no information about the context of instrument. The final version from the backward translation and the original English version was compared to ensure that there was no different meaning of each item of the instrument. Finally, the Indonesian version of EORTC QLQ-BR45 was field-tested in a pilot group consisting of 20 Indonesian breast cancer survivors to evaluate the translation quality and the practical aspects of test administrations. Each item was given to the participants to read and react to based on their understanding. The participants in the phase of psychometric evaluation were not included in the sample for this pilot study (49, 50). After pilot testing, the latest version of instrument was approved, and the next step was initiated.

### Phase 2: Psychometric evaluation

According to factor analysis, the sample size for this step was determined by counting the number of times each item appeared on the instrument with at least five, or 10 out of fifteen (51). We had to recruit an extra 10% of participant withdrawal because the EORTC QLQ-BR45 consisted of 45 items; thus, the sample size in this study ranged from 225 to 675 in total. Finally, a total of 635 of breast cancer patients were drawn from the Oncology Department of Dr. Moewardi Hospital and Dr. Kariadi Hospital. The two hospitals were selected as study locations because they are cancer referral center hospitals accessed by patients from various regions and are located on Java Island. Based on the results of the Indonesian population census in 2021, Java Island is inhabited by more than 50% of Indonesia's population and is the island with the highest population density compared to other islands in Indonesia.

Using the purposive sampling method, participants from the Department of Obstetrics and Gynecology of Moewardi Hospital, Surakarta, and the Oncology Ward of Dr. Kariadi Hospital Semarang, Central Java, Indonesia, were recruited from July 2021 to September 2021. The participants were chosen with certain criteria; namely, they had to be at least 18 years old who had a diagnosis of breast cancer (based on histological, cytological, or pathological findings), were able to read and understand the Indonesian language, and were willing to participate in the study. There were no restrictions regarding the Karnofsky Performance Scale (KPS) or comorbidities. Patients with psychiatric illness, cognitive impairment, or a diagnosis of another type of breast cancer were excluded. Participants were interviewed when they came for follow-up consultations at the outpatient clinic. Prior to interviewing breast cancer patients, the research team received permission and assist from clinicians in order to select suitable participants. The researchers then contacted the subject to explain the study and get written agreement. Four sets of questionnaires (EORTC QLQ-C30, EORTC QLQ-BR45, and sociodemographic data) would be completed in Indonesian by participants. Participants had the option of participating in face-to-face interviews with researchers or completing a questionnaire on their own. It took ~20 min to complete the questionnaire.

### Study instrument

The Indonesian version of EORTC QLQ-BR45, is an instrument that aims to measure the QoL among Indonesian breast cancer patients. The EORTC QLQ-BR45 is a supplementary questionnaire module that specifically measures the quality of life of breast cancer patients, to be used in conjunction with the EORTC QLQ-C30 core questionnaire. It consists of 45 items divided into nine multi-item subscales and three single items representing various aspects of quality of life among breast cancer patients. The multi-item scales include three functional scales (body image, sexual function, and breast satisfaction) and six symptom scales (systemic therapy side effects, arm symptoms, breast symptoms, endocrine therapy, skin mucosis symptoms, and endocrine sexual symptoms). Additionally, three single items measure the future impact on the perspective, sexual enjoyment, and upset due to hair loss. All items are rated on a 4-point Likert scale, ranging from *not at all* to *very much*. According to the EORTC QLQ-BR45 manual scoring, the raw score in EORTC QLQ-BR45 is linearly transformed to a scale of 0–100 points.

To measure the convergent validity, the EORTC QLQ-C30 as a questionnaire that measures the quality of life of cancer patients in general, was also used in this study. This questionnaire is already translated and validated in the Indonesian version (19). The EORTC QLQ-C30 consisted of 30 items, which were distributed across five multi-item functional scales (physical function, role function, emotional function), three multi-item symptom scales, one multi-item global health status, and six single-item scales on additional symptom reported by cancer patients. The questions in this questionnaire are rated on a 4-point Likert scale, with a total score ranges from 0 to 100 based on the EORTC QLQ-C30 manual book scoring.

In addition, our instrument also included seven items on health-related sociodemographic including age, education, marital status, monthly income, religion, occupation, and financial difficulties. Information on comorbidities, Karnofsky Performance Scale (KPS)-based cancer patient performance status score, and cancer stage of each patient were also collected from patient's medical record based on the most recent visit made by the patient.

### Statistical analysis

Patient characteristics were summarized using descriptive statistics. Mean and standard deviation were used for continuous variables while counts and percentages were used for categorical data.

An unweighted least square confirmatory factor analysis (CFA) was used to fit the measurement model. Model fit was assessed using the cumulative fit index (CFI), adjusted goodness of fit index (AGFI), root-mean-square error of approximation (RMSEA), and Tucker-Lewis index (TLI). A model with TLI, CFI (52), GFI (53), and AGFI > 0.9 (54) and RMSEA < 0.08 was also used (55). We also employed *X*^2^ statistics, which is actually a poor indicator of measurement model fit, for convention reason. Bartlett's test of sphericity and the Kaiser Meyer-Olkin (KMO) were used to measure the adequacy of sampling to be used as further evidence in proceeding to the construct validity analysis stage. The reference value for factor loading was 0.4, which represents at least a moderately strong correlation (56, 57).

To complement the results of the explanatory factor analysis (EFA) and CFA, the score of the convergent and discriminant validity was calculated. Convergent validity relates to the principle that measures of a construct should be highly correlated. Convergent validity occurs if the scores obtained from two different instruments measuring the same construct have a high correlation. For convergent validity, the Pearson correlation test was employed in this study to examine the relationship between the domain functional scale and the symptom scale on the Indonesian EORTC QLQ-BR45. The value of 0–0.30 represented negligible correlation; 0.30–0.50 indicated weak correlation; 0.05–0.70 represented moderately strong correlation; and >0.70 represented strong correlation (58–60).

Discriminant validity relates to the principle that measures of different constructs should not be highly correlated. Discriminant validity occurs when two different instruments measure two constructs that are predicted to be uncorrelated, resulting in a score that indeed shows non-correlation. The discriminant validity test was assessed based on the cross-loading measurement with the construct in this study.

Known-group validity was also measured to examine the instrument's capacity to differentiate the quality-of-life assessment items in early (stages I and II cancer) and late (stages III and IV cancer) stage patient groups. In addition, known-group validity was also used to evaluate the instrument's capacity for two groups of breast cancer patients with relatively good health (KPS ≥ 80%) and those with poorer health (KPS ≤ 70%). KPS of ≥80% reflects cancer patients who are able to do normal activities, and patients rated ≤70% are patients who need help from others and/or need treatment. An Independent *t*-test was used to investigate potential differences in the mean scale scores of the Indonesian EORTC QLQ-BR45 instrument between the groups.

Internal consistency reliability was evaluated using the Cronbach's alpha, and acceptable reliability was set at alpha >0.7 for all subscales (61). The R statistic package (version 2022.07.0) was employed in this study. A significance level of 0.05 was used throughout all inferential analyses.

## Results

### Participant characteristics

Six hundred and thirty-five breast cancer patients were included in this study with a response rate of 99%. To summarize, the median age of the participants was 52, with a range of 19–82 years old. Most participants (70.73%) were Muslim and 94.63% of participants had early-stage cancer. Table 1 shows the participants' characteristics in this study.

**Table 1:** Participant characteristics (*N* = 635).

**Characteristics**	***n***	**%**
**Age (years)**		
Median age−52; Range−19–82		
Mean ± standard deviation = 52.03 ± 9.58		
< 52	312	49.13
≥52	323	50.87
**Educational status**		
No formal education	35	5.51
Elementary school	189	29.76
Junior high school	122	19.21
Senior high school	193	30.39
Higher education degree	96	15.12
**Marital status**		
Married	615	96.85
Single/widowed/separate/discovered	20	3.15
**Monthly income**		
< 2,500,000 IDR^*^ (< 175.68 USD)	453	28.66
≥2,500,000 IDR^*^ (≥175.68 USD)	182	71.34
**Religion**		
Muslim	468	73.70
Christianity	163	25.67
Others	4	0.63
**Occupation**		
Unemployed	332	52.28
Farmer	46	7.24
Trader	146	22.99
Laborer	74	11.65
Government/official/enterprise/business	37	5.83
**Health insurance**		
BPJS (Indonesian universal coverage)	633	99.69
Private health insurance	2	0.31
**Stage of tumor**		
Early stage (stage I &II)	406	63.94
Late stage (stage III &IV)	229	36.06
**Cancer-comorbidity**		
Yes	116	18.27
No	519	81.73
**Financial difficulties**		
Insufficient and in debt	12	1.89
Sufficient and no saving	480	75.59
Sufficient and with saving	143	22.52
**Hospital**		
Dr. Kariadi Semarang Hospital	302	47.56
Dr. Muwardi Surakarta Hospital	333	52.44
**Karnofsky performance status**		
≤ 70%	504	79.37
≥80%	131	20.63

* *p* < 0.05.

### Adaptation

After collecting feedbacks from participants during the adaptation phase, three questions were corrected or further explained to all subjects in this study. The three items that underwent adjustment of word selections and sentence structure were: (1) Have you had hot flushes? (2) Have you had dry vagina? and (3) Have you been satisfied with the cosmetic result of the surgery? For example, researchers explained in more detail the word “hot flushes” with additional explanations such as, “do you feel hot and sweating 10–30 min after therapy?” This additional explanation is important to make patients understand the concept and data collection on the side effects of medicines given to the breast cancer patients can be done more easily and accurately. For vaginal dryness, explanatory questions or items such as “Does your vagina produce less vaginal discharge when stimulated by your partner?” were added. As for the item of “cosmetic result of the surgery,” an additional explanation was provided by asking question such as “Are you satisfied with the result of the mastectomy surgery?” This additional explanation will not change the meaning of the items in the original instrument. This was done because Indonesian has limited vocabularies if compared to English.

### Construct validity

**Figure 2:**
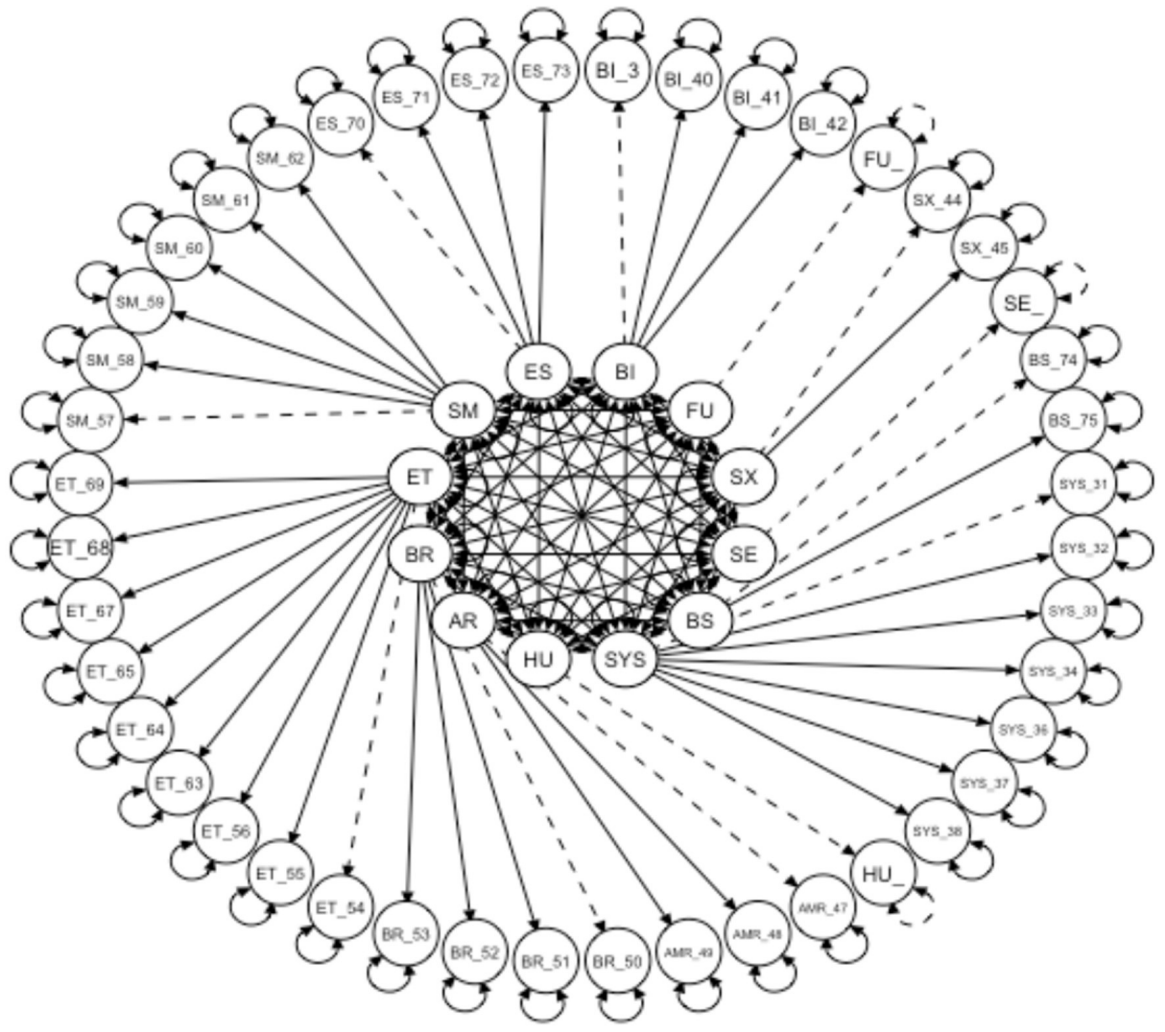
Confirmatory factor analysis model of the Indonesian EORTC BR-45 instrument.

The Indonesian version of the instrument for measuring QoL for breast cancer was translated from the original English instrument known as EORTC QLQ-BR45. A total of 635 participants with a mean age of ≥52.03 ± 9.58 participated in this study. The instrument consisted of 45 items distributed across eleven domains and fit an unweighted least square confirmatory factor analysis. The measurement model for the confirmatory factor analysis (CFA) is shown in Figure 2. The goodness of fit of the Indonesian version QLQ breast cancer was adequate based on the predetermined fit criteria, namely: χ^2^/df = 2,964.379, df = 839; CFI = 0.92; TLI = 0.90; GFI = 0.92; AGFI = 0.93; RMSEA = 0.06 (95% CI = 0.063–0.066); SRMR = 0.07. The adequacy index of sampling was 0.8 and the Bartlett's sphericity test was statistically significant (χ^2^ = 16,729.62; df = 990; *p*-value = < 0.001). All items in the model were loaded substantially on their respective factors, except for the items of the factor-constraint that could not be tested for its significance (Table 2). Table 2 shows that all correlation coefficient values between items and the domain themselves are 0.40, except for items no. 37, 54, 69, and 58. Finally, the measurement model was showed in Figure 2.

**Table 2:** Standardized factor loading of the Indonesian EORTC QLQ BR-45.

**Domain**	**No item/s**	**Factor loading**								
		**1**	**2**	**3**	**4**	**5**	**6**	**7**	**8**	**9**
**Body image (bi)**										
Felt physically less attractive	39	0.89								
Felt less feminine	40	0.88								
Have problem when naked	41	0.93								
Dissatisfied own body	42	0.81								
**Sexual functioning (sx)**										
Interested in sex	44		0.96							
Sexually active	45		0.96							
**Breast satisfaction (bs)**										
Satisfied with the cosmetic result of surgery	74			0.85						
Satisfied with the appearance of the skin	75			0.91						
**Systemic therapy side effects (sys)**										
Dry mouth	31				0.47					
Food and drink tasted different	32				0.66					
Eye painful	33				0.39					
Loss hair	34				0.59					
Feel unwell	36				0.67					
Hot flushes	37				**0.37**					
Headaches	38				0.49					
**Arm symptoms (arm)**										
Pain in arm or shoulder	47					0.78				
Swollen arm or hand	48					0.67				
Problem when raising arm or moving sideway	49					0.77				
**Breast symptoms (br)**										
Pain in area affected breast	50						0.70			
Swollen in affected breast	51						0.57			
Oversensitive in affected breast	52						0.79			
Skin problem	53						0.55			
**Endocrine therapy symptoms (et)**										
Sweated excessively	54							**0.23**		
Having mood swings	55							0.45		
Dizzy	56							0.49		
Having joints problem	63							0.74		
Stiffness in joints	64							0.76		
Joints pain	65							0.74		
Aches or pain in bones	66							0.81		
Aches of pain in muscles	67							0.78		
Gained weight	68							0.08		
Problem with gained weight	69							**0.15**		
		**1**	**2**	**3**	**4**	**5**	**6**	**7**	**8**	**9**
**Skin mucosis symptoms (sm)**										
Soreness in mouth	57								0.47	
Redness in mouth	58								**0.35**	
Pain in hand or feet	59								0.71	
Redness in hands and feets	60								0.39	
Tingling in fingers or toes	61								0.48	
Numbness in fingers or toes	62								0.67	
**Endocrine sexual symptoms (es)**										
Dry vagina	70									0.93
Discomfort with vagina	71									0.96
Pain in vagina during sexual activity	72									0.42
Experienced a dry vagina during sexual activity	73									0.45

The value in bold indicates the absolute value of the loading factor where the value is < 0.40. All values in each domain are significant.

### Known-group validity

To examine the known-group validity of the Indonesian EORTC QLQ-BR45, the scores were compared across patients with different disease stages (stages I–II vs. stages III–IV) and according to the KPS (Table 3). The results provided evidence that the scores of the five of the multi-item scale (body image, breast satisfaction, systemic therapy, arm symptom, breast symptom, endocrine therapy system, and endocrine sexual symptom) were significantly different for both KPS and disease stage. For example, patients with a KPS of ≥80% did better than those with a KPS *of* ≤ 70%. The same was also true for two multi-item scales of functional scales (body image and breast satisfaction) and five multi-item of symptom scales (systemic therapy side effects, arm symptom, breast symptom, endocrine therapy symptom, and endocrine sexual symptom). According to the results of the functional scale of the quality of life for breast cancer, the score of body image among patients with late-stage breast cancer was significantly higher than that of patients in early stage (Stage III & IV). This pattern was also found in the three multi-item scale of symptoms scale (systematic therapy side effect, arm symptom, and breast symptom) where the score was significantly higher among late-stage patients compared to patients in early stage.

**Table 3:** Known-group differences by Karnofsky performance status and disease stage.

**Domain**	**KPS**			**Disease stage**		
	**KPS** ≤ **70%**	**KPS** ≥**80%**		**Stage I–II**	**Stage III–IV**	
	**Mean (SD)**	**Mean (SD)**	***p*** **-value**	**Mean (SD)**	**Mean (SD)**	***p*** **-value**
Body image	5.56 (2.58)	6.22 (3.44)	0.014	5.65 (2.71)	5.79 (2.94)	0.014
Sexual functioning	3.50 (1.82)	3.46 (1.90)	0.504	3.46 (1.89)	3.46 (1.89)	0.358
Breast satisfaction	6.73 (2.05)	6.71 (1.95)	0.001	6.73 (2.05	6.71 (1.95)	0.001
Systemic therapy side effects	12.92 (4.62)	15.05 (4.65)	0.050	13.69 (4.95)	13.69 (4.95)	0.001
Arm symptoms	5.12 (2.39)	5.95 (2.74)	0.001	5.12(2.39)	5.95 (2.74)	0.0001
Breast symptoms	6.51 (2.72)	7.74 (3.00)	0.001	6.51 (2.72)	7.74 (3.00)	0.0001
Endocrine therapy symptoms	15.10 (4.64)	16.01(5.55)	0.050	15.36 (4.91)	15.13 (4.74)	0.587
Skin mucosis symptoms	8.86 (3.24)	9.21 (2.84)	0.188	8.84 (3.24	9.21 (2.84)	0.188
Endocrine sexual symptoms	5.85 (2.53)	4.47 (1.39)	0.001	5.53 (2.43)	5.64 (2.35)	0.610

### Convergent validity

In terms of convergent validity, the coefficient correlation of the functional scale and symptom scale between the Indonesian EORTC QLQ-BR45 and EORTC QLQ-C30 was significantly adequate. Table 4 shows the results of the convergent validity.

**Table 4:** Convergent validity on Indonesian EORTC QLQ BR45 (*n* = 635).

**Dimension**	**Functional scale**			**Symptoms scale**		
	**Mean (SD)**	***r***	**95% CI**	**Mean (SD)**	***r***	**95% CI**
Indonesian version of EORTC QLQ BR-45	19.56 (4.99)	0.581	0.53–0.63^***^	58.63 14.21)	0.958	0.951–0.96^***^
EORTC QLQ 30	38.95 (8.07)	0.908	0.89–0.92^***^	28.97 (2.91)	0.510	0.455–0.569^***^

*** *p* < 0.001.

### Inter-factor correlation

The inter-factor correlations of the Indonesian EORTC QLQ-BR45 subscales are presented in Table 5. All subscales of this instrument were low, but most of them were significant. The small magnitude of those correlations revealed the state of overpowered. The body image subscale was positively associated with seven multi-item subscales of this instrument (sexual function, systemic therapy side effect, arm symptom, breast symptom, endocrine therapy symptom, skin mycosis symptom, and endocrine sexual symptom), while the subscale of breast satisfaction was excluded. The subscale of breast symptoms was negatively associated with three subscales of body image, breast symptom, and endocrine sexual symptom.

**Table 5:** Inter-factor correlation of Indonesian EORTC QLQ BR-45 subscale scores.

**Domain**	**Body image**	**Sexual function**	**Breast satisfaction**	**Systemic therapy side effects**	**Arm symptom**	**Breast symptom**	**Endocrine therapy symptom**	**Skin mucosis symptom**	**Endocrine sexual symptom**
Body image		0.149^***^	−0.133^***^	0.363^***^	0.243^***^	0.232^***^	0.366^***^	0.235^***^	0.169^***^
Sexual function	0.149^***^		−0.041	−0.004	−0.081^*^	−0.022	−0.017	−0.039	0.087^**^
Breast satisfaction	−0.133^***^	−0.041		0.008	−0.040	−0.108^**^	0.015	0.061	−0.108^**^
Systemic therapy side effect	0.363^***^	−0.004	0.008		0.405^***^	0.243^***^	0.458^***^	0.418^***^	0.002
Arm symptom	0.243^***^	−0.082^*^	−0.040	0.405^***^		0.453^***^	0.338^***^	0.348^***^	0.007
Breast symptom	0.232^***^	−0.022	−0.022	0.243^***^	0.453^***^		0.187^***^	0.241^***^	0.045
Endocrine therapy symptom	0.366^***^	−0.017	0.015	0.458^***^	0.338^***^	0.187^***^		0.531^***^	0.109^**^
Skin mucosis symptom	0.235^***^	−0.039	0.061	0.418^****^	0.348^***^	0.241^***^	0.530^***^		0.069
Endocrine sexual symptom	0.169^***^	0.090^*^	−0.108^**^	0.021	0.007	0.045	0.109^**^	0.069	

*** p < 0.001,

** p < 0.01,

* p < 0.05.

### Internal consistency

The internal consistency of the Indonesia EORTC QLQ-BR45 was satisfactory with a Cronbach alpha of 0.87 for the overall scale. The Cronbach's alpha values for all domains are presented in Table 6, where it is shown to be ranging between 0.68 and 0.95. Table 6 shows that all domains have good internal consistency reliability with the exception of skin mucosis symptoms (Cronbach's alpha = 0.68).

**Table 6:** Cronbach's alpha of each domain of the Indonesian EORTC QLQ BR-45.

**Domain**	**Cronbach's alpha**	**95% CI**
**Functional scale**		
Body image	0.93	0.92–0.94
Sexual functioning	0.95	0.94–0.96
Breast satisfaction	0.95	0.94–0.95
**Symptom scale**		
Systemic therapy side effects	0.72	0.69–0.75
Arm symptoms	0.77	0.74–0.80
Breast symptoms	0.74	0.71–0.77
**Target therapy scale**		
Endocrine therapy symptoms	0.73	0.70–0.76
Skin mucosis symptoms	0.68	0.63–0.71
Endocrine sexual symptoms	0.79	0.77–0.82

## Discussion

This study was conducted with the aim of performing cultural adaptation of the original EORTC QLQ-BR45 instrument in English into the Indonesian version of the instrument, and assessing its psychometric properties. To the best of our knowledge, this is the first translation and application of a valid Indonesian version of EORTC QLQ-BR45 in breast cancer context in Indonesia. By following the forward-backward translation method recommended by the international guideline for EORTC research group, a rigorous approach was applied to translate the original English version of the EORTC QLQ-BR45 (35, 47, 62) into a validated and culturally sensitive simplified Indonesian version. The finding of the first phase showed that there is a linguistic and conceptual equivalence between the Indonesian version of EORTC QLQ-BR45 measurement and the original English version, thereby ensuring good content validity. When an instrument is to be utilized across cultures, not only should it be correctly translated linguistically, but it should also be culturally adapted to ensure conceptual content validity (62). In addition, the field test of this phase's instrument revealed the necessity for modest modifications, such as the replacement of difficult-to-understand words and phrases (for example on the questions “Have you had hot flushes?;” “Have you had dry vagina?,” and “Have you been satisfied with the cosmetic result of the surgery?”). Thus, further explanations from the researchers to participants are needed. This is in line with previous studies which showed that filling out the questionnaire took a considerable amount of time due to the presence of non-medical research participants and the impact of their cognitive deterioration (63). However, all breast cancer patients in the pilot study were able to provide responses for all items.

Furthermore, the result of the psychometric testing showed good and is valid to be used for Indonesian breast cancer patients as it has high acceptability and comprehensibility. Our results confirm that this instrument is structurally comprised of a function scale (body image, sexual function, and breast satisfaction); a symptom scale (systemic therapy side effect, arm symptom, and breast symptom); and a target therapy scale (endocrine therapy symptom, skin mycosis symptom, and endocrine sexual symptom). Those final models correspond to the construct domains of the original version of the EORTC QLQ-BR45 (35). All items selected in the new instrument version greatly contribute to the factor, as they have satisfactory factorial loading for developing the construct of this instrument.

In addition, the internal consistency reliability of the Indonesian version of the instrument and its subscale is good, and we have demonstrated a strong internal consistency for the Indonesian EORTC QLQ-BR45. Notably, all domains are acceptable, which is in line with the previous study (35). However, the skin mucosis system scale has a low internal consistency coefficient, which is similar to the finding in a previous study on Bahraini (64) and Moroccan survivors of breast cancer (65). The higher internal consistency of the instrument in this study, when compared to different cultural contexts, is represented by strong evidence of reliability, showing that this tool is well-designed and qualified for a short response time as indicated by comments from all participants. This document confirms that the Indonesian EORTC QLQ-BR45 is meaningful and valuable for Indonesian breast cancer survivors.

Regarding the dimensionality, some items are not loaded in their original domains, such as hot flashes, excessive sweats, soreness in the mouth, and weight gain. These could be due to the uncommon symptoms experienced by cancer patients. For example, it is uncommon that cancer patients treated with chemotherapy and/or radiotherapy to experience weight gain. In a previous study, it is reported that hot flashes occur frequently and are associated with unpleasant symptoms and poor quality of life in breast cancer patients (66). The specific symptoms, due to the specific nature of the questionnaire, also become the reasons for the factors to be loaded. In the previous study, the factors are loaded until the second factor because the questionnaire is more general (19). The low correlation between items and their factors could be caused by the uncommon symptoms experienced by the patients.

The significant differences in domain scores, based on the KPS and cancer stage, are shown by body image, breast satisfaction, arms symptoms, and breast symptoms. This instrument can well-distinguish cancer patients with different stages. Moreover, the Indonesian EORTC QLQ-BR45 instrument can differentiate sexual function, endocrine system therapy, and skin mucosis symptoms based on the KPS and levels. This may link to the possibility of the lack of sexual activities among cancer patients in all cancer stages; the hormonal treatment provided to the patients with all KPS and all cancer stage; and skin mucosis symptoms that are mostly experienced by the patients.

The inter-factor correlation shows that sexual function, breast satisfaction, and endocrine sexual function mostly have insignificant correlations. In a previous study involving 65 French patients during an adjuvant endocrine therapy, 60% of the patients reported that they experience sexual problems (67). This is because women who undergo mastectomy tend to have a feeling of inferiority in terms of their body image (68). Another reason is that it can be assumed that cancer patients have difficulties in answering questions related to the three functions (69), which can be due to the uncommon symptoms or maybe the privacy nature of the questions that make patients feel insecure in answering them, especially for items related to sexual activities. Based on a previous study, most participants often feel uncomfortable or embarrassed to answer sensitive questions, such as those related to sexual activities (11, 70). However, if the domains are categorized into functional and symptom scales, the correlation values are high. Good internal consistencies of all domains are also presented. Thus, the Indonesian EORTC QLQ-BR45 is still considered satisfactory for implementation in the Indonesian population.

## Strengths and limitation of this study

The large sample size becomes the strength of this study, as reflected in the result of KMO which shows that the sample size is calculated according to the rules and facilitates further analysis using the confirmatory factor analysis. To our knowledge, this study is the first validation study on Indonesian breast cancer patients during therapy with strong overall internal consistency and reliability. We have also established that the Indonesian EORTC QLQ-BR45 is strongly indicative of the level of functional scale and symptom scale among breast cancer patients. In the future, this instrument can be used to measure the quality of life of breast cancer patients during treatment and evaluate the therapy given by doctors, which ultimately will improve the quality of life of these patients and may prolong their survival.

Despite the strength of this study, some limitations are identified. First, as in most studies, sexual activity aspect is one of the aspect affected by the long-lasting series of adjuvant breast cancer therapy, and this will affect the personal relationship between the cancer patients and their husband or partner (71–73). In this study, there is no measure of sexual activity/function before diagnosis, and the impact of cancer diagnosis and treatment on sexual activity and function cannot be examined due to the cross-sectional design of the study. Second, all psychometric analyses were performed on the same sample in the same data set, where the preference is to do them in separate samples and data sets. However, the KMO results reflects the high adequacy of the sample for further analysis to measure construct validity.

## Conclusion

In conclusion, the Indonesian EORTC QLQ-BR45 is a product of a translation process that is performed according to the standard process for translating instruments, consisting of translation and back-translation, pilot testing, and a validation study. The scale is valid, acceptable, and has good psychometric properties. The Indonesian EORTC QLQ-BR45 has the ability to distinguish breast cancer patients with different levels of functional scale and symptom scale. So far, health services only focus on reducing the symptoms during breast cancer therapy. Through the use of this instrument, healthcare professionals can design evidence-based and personality therapies for patients with advanced malignancies, including breast cancer.

## Data availability statement

The original contributions presented in the study are included in the article/supplementary material, further inquiries can be directed to the corresponding author.

## Ethics statement

The studies involving human participants were reviewed and approved by Research Ethics Committee of Ahmad Dahlan University, Yogyakarta, Indonesia (No. 012102016). The patients/participants provided their written informed consent to participate in this study.

## Author contributions

SS and DP: conceptualization, formal analysis, and investigation. SS, DP, and DR: methodology. SS: project administration and funding acquisition. DP: data curation and supervision. All authors: drafting, writing, reviewing, and editing. All authors contributed to the article and approved the submitted version.
